# Retraction: De Vecchis, R., et al. Platypnea-Orthodeoxia Syndrome: Multiple Pathophysiological Interpretations of a Clinical Picture Primarily Consisting of Orthostatic Dyspnea. *J. Clin. Med.* 2016, *5*, 85

**DOI:** 10.3390/jcm10040883

**Published:** 2021-02-22

**Authors:** Renato De Vecchis, Cesare Baldi, Carmelina Ariano, JCM Editorial Office

**Affiliations:** 1 Cardiology Unit, Presidio Sanitario Intermedio “Elena d’Aosta”, ASL Napoli 1 Centro, 80137 Napoli, Italy; carmelariano@tiscali.it; 2 Heart Department, Interventional Cardiology, Azienda Ospedaliero-Universitaria “San Giovanni di Dio e Ruggi d’Aragona”, 84131 Salerno, Italy; giovenale80@gmail.com; 3 MDPI, St. Alban-Anlage 66, 4052 Basel, Switzerland; jcm@mdpi.com

The journal retracts the article “Platypnea-Orthodeoxia Syndrome: Multiple Pathophysiological Interpretations of a Clinical Picture Primarily Consisting of Orthostatic Dyspnea” by De Vecchis, R., et al. [1].

Following publication, concerns were brought to the attention of the publisher regarding an alleged redundant publication with “Platypnea-orthodeoxia Syndrome: Orthostatic Dyspnea and Possible Pathophysiological Substrates” by De Vecchis, R., et al. [2]. 

Adhering to our complaints procedure, an investigation was conducted that confirmed the existence of a redundant publication and the article is therefore retracted.

This retraction was approved by the Editor-in-Chief of Journal of Clinical Medicine.

The authors agreed to this retraction. 
